# Sonophysical mechanisms of bubble fragmentation near an ultrasonically vibrating boundary: influence of frequency and amplitude on interfacial intensification

**DOI:** 10.1016/j.ultsonch.2026.107852

**Published:** 2026-04-10

**Authors:** Zhitao Zhao, Wei Lu, Qingsong Zhang, Hui Zhuo, Zhaoyang Su

**Affiliations:** a School of Safety Science and Engineering, Anhui University of Science and Technology, Huainan 232001, China; b National Key Laboratory of Safe Mining of Deep Coal and Environmental Protection, Anhui University of Science and Technology, Huainan 232001, China; c Anhui Panshi Mining Technology Co., Ltd, Huainan 232001, China

**Keywords:** Ultrasonic cavitation, Bubble fragmentation, Sonophysical effects, Microjet dynamics, Vibrating boundary, Process intensification

## Abstract

Ultrasonic cavitation has been increasingly employed to intensify wet dust removal by enhancing gas-liquid interfacial renewal and local mixing. However, the coupled effects of ultrasonic frequency and vibration amplitude on bubble collapse dynamics, fragmentation behavior, and cavitation sustainability remain insufficiently quantified. In this study, a numerical investigation is conducted to elucidate the collapse and breakup characteristics of a millimeter-scale single bubble near an ultrasonically vibrating wall, representing a typical cavitation-assisted scrubbing environment. A two-phase Volume of Fluid (VOF) model is used to resolve transient interface deformation, asymmetric collapse, and microjet formation under ultrasonic excitation. The effects of ultrasonic frequency (20–40 kHz) and vibration amplitude (5–30 μm) on bubble morphology, air volume fraction evolution, flow-field velocity, and fragmentation degree are systematically examined. The results show that collapse is dominated by directional contraction toward the vibrating boundary and the formation of high-speed penetrating microjets, which govern bubble breakup and interfacial generation. Increasing frequency and amplitude intensifies fragmentation and jet impact strength; however, excessive excitation promotes rapid depletion of residual bubble structures and suppresses sustained cavitation activity. Within the investigated range, the combination of 25 kHz and 20 μm provides a favorable balance between collapse intensity and cavitation persistence, enabling sustained fragmentation and enhanced interfacial complexity. Our study offers mechanistic insights and parameter-selection guidance for ultrasonic cavitation-assisted intensification of wet dust scrubbing and related gas-liquid-solid processes.

## Highlights

• Sonophysical mechanisms of bubble fragmentation near a vibrating wall were investigated.
• Microjet formation drives bubble breakup and enhances interfacial renewal.
• Frequency modulates the balance between collapse intensity and cavitation persistence.
• Excessive excitation leads to rapid nuclei depletion and cavitation activity decay.
• A favorable operating condition was identified within the investigated frequency–amplitude range.

## Graphical abstract

**Figure:**
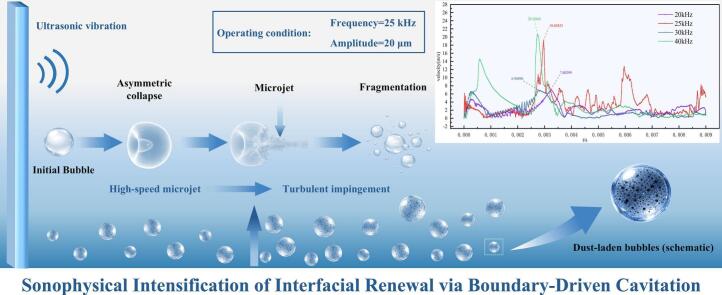

## 1 Introduction

In multi-phase engineering systems, particularly those involving high-concentration particulate matter in confined environments, the efficiency of interfacial mass transfer remains a fundamental bottleneck [1], [2], [3]. Conventional wet scrubbing techniques, while widely adopted for their intrinsic safety, often suffer from limited gas-liquid interfacial area and slow interfacial renewal rates [4], [5], [6]. These limitations stem from the stable boundary layers surrounding fine particles and bubbles, which inhibit effective collision and capture kinetics [7], [8], [9]. Consequently, sonophysical intensification strategies—capable of disrupting these boundary layers and inducing high-frequency interfacial disturbances—have emerged as a pivotal research frontier in advanced process intensification [10], [11].

Ultrasonic cavitation, characterized by the nucleation, nonlinear oscillation, and violent collapse of microscopic bubbles, provides a robust pathway for sonomechanical intensification [12]. Sonophysical and sonochemical mechanisms should be distinguished in ultrasonic cavitation processes. Sonophysical effects mainly involve bubble oscillation, asymmetric collapse, microjet formation, local shear enhancement, and interfacial renewal, whereas sonochemical effects are generally associated with radical generation and chemically reactive species produced under extreme cavitation conditions[13], [14]. The physics of bubble collapse involves a rapid conversion of acoustic energy into localized mechanical work, manifested as high-speed microjets, spherical shock waves, and intense turbulent dissipation [15], [16]. In the context of gas-liquid-solid systems, these phenomena induce strong shear stress gradients and micro-streaming, which drastically enhance local mixing and promote particle-interface interactions such as collision and adhesion [17], [18]. While the potential of sonochemical and sonophysical effects in particle separation and surface cleaning is well-recognized [19], [20], the underlying dynamics are governed by highly non-linear, multiscale fluid processes that are sensitive to the acoustic pressure topology [21], [22], [23], [24], [25].

A critical yet under-explored aspect of cavitation dynamics is the coupling between the bubble and an active vibrating boundary. Previous studies have shown that bubbles near solid boundaries under ultrasonic excitation can undergo asymmetric collapse, jet penetration, and strong wall-directed deformation[26]. These studies provide important insight into the general microjet dynamics of near-wall bubbles. However, the fragmentation behavior of a representative bubble near an actively vibrating boundary, and its implication for interfacial renewal in ultrasonic-assisted gas-liquid systems, remain insufficiently understood. Most existing studies focus on bubble behavior near static rigid walls [27], [28]; however, in many sonochemical reactors and intensification devices, the cavitation occurs in the immediate vicinity of the vibrating transducer surface. This active boundary introduces a time-varying displacement and a localized, high-gradient acoustic field, significantly altering the Bjerknes forces and the symmetry of bubble contraction [29], [30], [31], [32], [33]. Resolving these interactions is numerically challenging due to the extreme separation of scales—ranging from millimeter-scale bubble motion to microsecond-scale jet formation—and the stringent stability requirements of high-frequency boundary conditions [34], [35].

Despite the extensive literature on cavitation bubble dynamics, most previous studies have primarily focused on micron-scale cavitation nuclei and their collapse behavior near rigid boundaries or in ideal acoustic fields [36], [37]. However, in ultrasonic-assisted wet dust-removal systems, the gas phase typically exists in the form of millimeter-scale dust-laden bubbles, whose dynamic response to ultrasonic excitation differs fundamentally from that of classical cavitation nuclei [38], [39].

In such systems, the interaction between a vibrating boundary and a macroscopic bubble governs processes such as bubble deformation, breakup, and interfacial renewal, which are directly related to gas-liquid mass transfer and particle capture efficiency. Nevertheless, the fragmentation behavior of these bubbles under different ultrasonic conditions, as well as the balance between collapse intensity and post-collapse persistence, remains insufficiently understood from a sonophysical perspective. Therefore, the present study focuses on the ultrasonic-induced deformation, asymmetric collapse, and fragmentation of a representative millimeter-scale bubble near an actively vibrating boundary. By systematically analyzing the effects of frequency and vibration amplitude, this work aims to clarify the underlying mechanisms governing bubble breakup and interfacial intensification, and to provide mechanistic insight and useful guidance for ultrasonic-assisted wet dust-removal processes.

## 2 Numerical model and computational Methods

### 2.1 Idealized physical model of boundary-driven ultrasonic cavitation

**Fig. 1:**
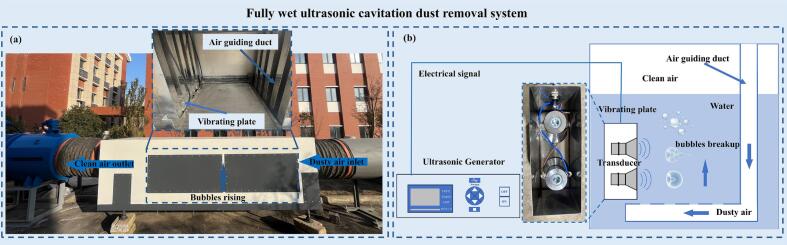
Fully wet ultrasonic-assisted dust-removal system. (a) Photograph of the developed device and the local enlarged structure of the air-guiding duct and vibrating plate. (b) Schematic of the ultrasonic action zone, showing the interaction between the vibrating boundary and rising dust-laden bubbles.

In this study, ultrasonic cavitation is introduced as a physical intensification approach into a wet dust-removal process for controlling hazardous respirable dust in underground environments. A fully wet ultrasonic cavitation-assisted dust-removal system is designed (Fig. 1), in which cavitation-induced bubble collapse is employed to enhance gas-liquid interfacial interactions and improve dust capture efficiency.

An axial-flow induced-draft fan generates a stable negative pressure within the system. Dust-laden air is drawn into the device and guided to the bottom of the water tank, where it is dispersed into numerous dust-laden bubbles through perforations in the guide duct (Fig. 1(a)). These bubbles provide the initial gas-liquid contact required for subsequent cavitation-enhanced interfacial processes.

Fig. 1(a) presents the developed device and the locations of its main functional components, including the dust-laden air inlet, clean-air outlet, bubble rising region, and the local structure composed of the air-guiding duct and vibrating plate. Dust-laden air enters the device through the inlet and is guided to the lower part of the water tank through the air-guiding duct, where it is dispersed into millimeter-scale dust-laden bubbles. These bubbles then rise through the liquid phase and undergo preliminary gas-liquid contact.

Fig. 1(b) is a simplified schematic of the local ultrasonic action zone corresponding to the enlarged structure shown in Fig. 1(a). In this region, the ultrasonic transducer drives the vibrating plate to generate a high-frequency excitation field near the wall. As the rising dust-laden bubbles pass through this zone, they undergo deformation, asymmetric collapse, and breakup near the vibrating boundary, thereby increasing gas-liquid interfacial renewal. The cleaned airflow finally accumulates above the liquid level and is discharged through the outlet.

To clarify the fundamental sonophysical mechanism underlying this process, the following numerical model focuses on the local interaction between a representative bubble and the vibrating boundary, rather than the entire multiphase flow in the full-scale device. The local enlarged structure in Fig. 1(a) corresponds to the ultrasonic action zone schematically illustrated in Fig. 1(b), where the interaction between the vibrating plate and rising dust-laden bubbles forms the physical basis of the bubble breakup mechanism investigated in this study.

### 2.2 Mathematical model and governing equations

The Volume of Fluid (VOF) two-phase flow model was adopted to numerically investigate the bubble collapse behavior induced by ultrasonic cavitation-driven turbulent impingement. In the cavitating flow field under ultrasonic excitation, the flow behavior is governed by the continuity equation, momentum equation, and energy equation. Thermal effects are weak under the investigated conditions, and the energy equation is solved to ensure numerical stability, while the temperature field remains approximately uniform[40]. These equations can be expressed as follows:

(1) $$\partial \rho_{m}/\partial t+\nabla \cdot \left(\rho_{m}v\right)=0$$

(2) $$\partial \left(\rho_{m}v\right)/\partial t+\nabla \cdot \left(\rho_{m}vv\right)=-\nabla p+\nabla \cdot \left[\mu_{m}\left(\nabla v+\nabla v^{T}\right)-2\mu_{m}\left(\nabla \cdot v\right)I/3\right]+{\vec{F}}_{\sigma }$$

(3) $$\partial \left(\rho_{m}E\right)/\partial t+\nabla \cdot \left[v\left(\rho_{m}E+p\right)\right]=\nabla \cdot \left(k\nabla T\right)$$

where p denotes the pressure field (Pa), and v is the velocity vector (m/s). vv denotes the dyadic product of the velocity vector. $\rho_{m}$(kg/m^3^) and $\mu_{m}$(Pa·s) represent the density and dynamic viscosity of the mixture respectively. *E* is the total energy per unit mass (J kg^–1^), T is the temperature (K), k is the thermal conductivity (W m^–1^ K^–1^), and *I* is the identity tensor.

The surface tension effect at the gas-liquid interface of the bubble is taken into account. According to the Continuum Surface Force (CSF) model proposed by Brackbill [41], surface tension is incorporated into the momentum equation as an equivalent volumetric force term. The surface tension force ${\vec{F}}_{\sigma }$ is expressed as

(4) $${\vec{F}}_{\sigma }=\sigma \kappa \nabla \alpha_{l}$$

Following the CSF model, surface tension is represented as a volumetric force acting at the gas-liquid interface. Here σ is the surface tension coefficient (N/m), $\alpha_{l}$ is the volume fraction of the liquid phase in a computational cell, and $\kappa =\nabla \cdot (\nabla \alpha_{l}/|\nabla \alpha_{l}|)$ denotes the curvature of the phase interface.

In the VOF method, the gas-liquid interface is reconstructed and tracked by solving the volume fraction of each phase in every computational cell at each time step [42]. The transport equation for the liquid-phase volume fraction α is given by

(5) $$\partial \alpha_{l}/\partial t+\nabla \cdot \left(\alpha_{l}v\right)=0$$

When$\alpha_{l}$ = 1, the cell is fully occupied by the liquid phase. When$\alpha_{l}$ = 0, the cell contains only the gas phase. Cells with 0＜$\alpha_{l}$＜1 correspond to interfacial cells containing both phases.

The density and dynamic viscosity of the mixture are calculated using a volume-fraction-weighted formulation:

(6) $$\rho_{m}=\alpha_{l}\rho_{l}+\alpha_{g}\rho_{g}$$

(7) $$\mu_{m}=\alpha_{l}\mu_{l}+\alpha_{g}\mu_{g}$$

where subscripts l and g denote the liquid and gas phases, respectively, and $\alpha_{g}=1-\alpha_{l}$ The liquid phase is treated as an incompressible Newtonian fluid with constant density. The gas phase is treated as compressible ideal air, and its density is calculated using the ideal gas equation of state:

(8) $$\rho_{g}=pM/\left(R_{u}T\right)$$

where *M* is the molar mass of air (kg mol^–1^) and $R_{u}$ is the universal gas constant (8.314 J mol^–1^ K^–1^).

### 2.3 Boundary conditions and numerical implementation

To investigate the collapse dynamics of a single bubble near an ultrasonically vibrating wall, the ultrasonic action region within the water tank was idealized based on the local structure shown in Fig. 1(b). The complex gas-liquid interaction process in practical dust-removal devices was simplified as the evolution of an initially spherical bubble in a liquid subjected to an ultrasonic field.

Ultrasonic excitation is imposed by prescribing a harmonic displacement boundary condition on the rigid wall:

(9) y(t)=Asin(2πft)

where A (μm) is the vibration amplitude of the ultrasonic transducer, f (Hz) is the ultrasonic frequency, and t (s) denotes time. Differentiating Eq. (9) with respect to time yields the corresponding velocity expression of the vibrating boundary:

(10) $$U_{w}\left(t\right)=2\pi fAcos\left(2\pi ft\right)$$

This formulation ensures that frequency-amplitude coupling directly governs the boundary-induced inertial forcing. All remaining walls are treated as no-slip boundaries. The far boundary is specified as a pressure outlet to minimize wave reflection. Gravity is neglected because the characteristic collapse timescale (microseconds) is much shorter than the buoyancy timescale for millimeter-scale bubbles.

Although bubble collapse is inherently three-dimensional, previous studies have demonstrated that two-dimensional simulations can capture the dominant features of asymmetric jet formation and interface deformation under near-axisymmetric conditions. The present two-dimensional formulation therefore aims to resolve the primary collapse mechanism with acceptable computational cost, while acknowledging that fine-scale three-dimensional instabilities may not be fully represented.

The flow is treated as transient. Pressure-velocity coupling is handled using the Pressure-Implicit with Splitting of Operators (PISO) algorithm [43]. Spatial discretization employs a second-order upwind scheme, and temporal discretization uses an implicit scheme to ensure numerical stability under high-frequency excitation. A time step of 5.0 × 10^-7^ s is adopted, corresponding to approximately 80 time steps per acoustic cycle at 25 kHz, which ensures sufficient temporal resolution of collapse dynamics.

To provide an equivalent macroscopic representation of flow disturbances induced by cavitation jets, the standard k–ε turbulence model is employed. The present study focuses on interface evolution and collapse asymmetry rather than detailed turbulence spectrum resolution; thus, the turbulence model serves as an effective closure for large-scale flow fluctuations.

### 2.4 Computational domain and grid independence

**Fig. 2:**
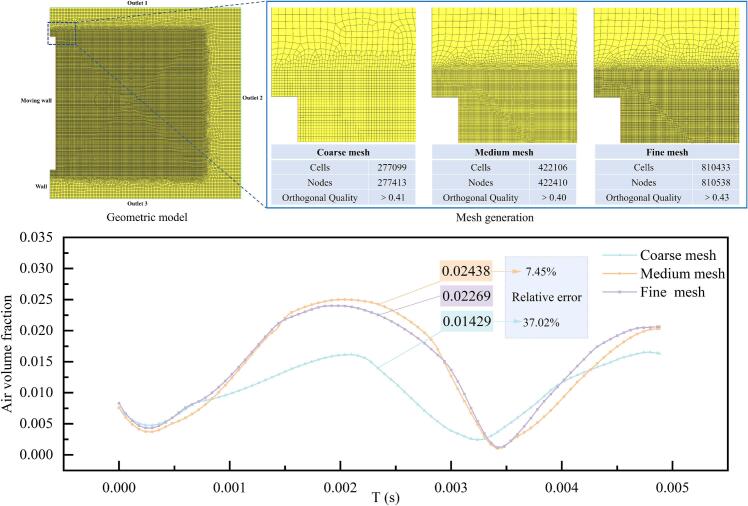
Computational geometry and mesh generation for the ultrasonic cavitation simulation, together with the mesh independence analysis. The upper panels show the computational domain and the coarse, medium and fine mesh distributions, while the lower panel compares the temporal evolution of air volume fraction used to assess mesh independence.

The ultrasonic cavitation device was simplified into a two-dimensional computational model, as shown in Fig. 2. The computational domain was defined as a square region. The initial bubble radius was set to 4 mm, and the initial coordinates of the bubble center were (30, 50) mm. Here, the 4 mm radius does not represent a microscopic cavitation nucleus. Instead, it is adopted as a simplified representative dust-laden bubble in the ultrasonic-assisted wet dust-removal system, so that its deformation, asymmetric collapse, jet penetration, and fragmentation near the vibrating boundary can be clearly resolved within the present VOF framework. To minimize the influence of pressure boundaries on bubble deformation and collapse, the size of the computational domain was set to 100 × 100 mm.

To verify mesh independence, three mesh resolutions were tested, referred to as a coarse mesh, medium mesh, and fine mesh respectively. For all cases, the minimum mesh quality was maintained above 0.40. Since bubble deformation and collapse induced by ultrasonic cavitation lead to variations in the air volume fraction within the flow field, the air volume fraction was selected as the evaluation criterion. The temporal evolution of the air volume fraction was monitored over a period of 0.005 s for the different mesh resolutions, as illustrated in Fig. 2.

The grid independence analysis indicates that the temporal evolution of the air volume fraction exhibits similar trends for all mesh resolutions. The results obtained using the medium and fine meshes show good agreement, with relative differences remaining within 10%. In contrast, the coarse mesh deviates significantly from the other two cases after 0.001 s. At approximately t≈0.0023 s, the relative error exceeds 37%, demonstrating that the coarse mesh does not provide sufficient accuracy.

Considering both computational accuracy and efficiency, the medium mesh was selected for all subsequent simulations. The adopted mesh consists of unstructured quadrilateral elements, with a total of 422,106 cells and 422,410 nodes. The minimum mesh quality is greater than 0.4, and the average mesh quality reaches 0.99.

## 3 Results and discussion

Numerical simulations were performed to investigate bubble collapse near an ultrasonically vibrating surface under different ultrasonic parameters. The ultrasonic frequencies considered were 20, 25, 30, and 40 kHz, while the vibration amplitudes were set to 5, 10, 20, and 30 μm. The initial bubble radius was fixed at 4 mm, and the initial distance between the bubble center and the transducer vibrating surface was set to 27 mm. The initial temperature of the flow field was 300 K, and both the initial internal bubble pressure and the surrounding liquid pressure were set to 0.1 MPa.

### 3.1 Morphological evolution of bubble collapse induced by ultrasonic cavitation jets

Within the selected ranges of ultrasonic frequency (20–40 kHz) and vibration amplitude (5–30 μm), a preliminary comparison of the overall bubble collapse processes indicates that the bubbles exhibit similar qualitative evolution patterns under ultrasonic cavitation. Regardless of the specific operating condition, the bubble dynamics are characterized by successive stages of deformation, asymmetric contraction, and cavitation jet impact when subjected to ultrasonic excitation.

**Fig. 3:**
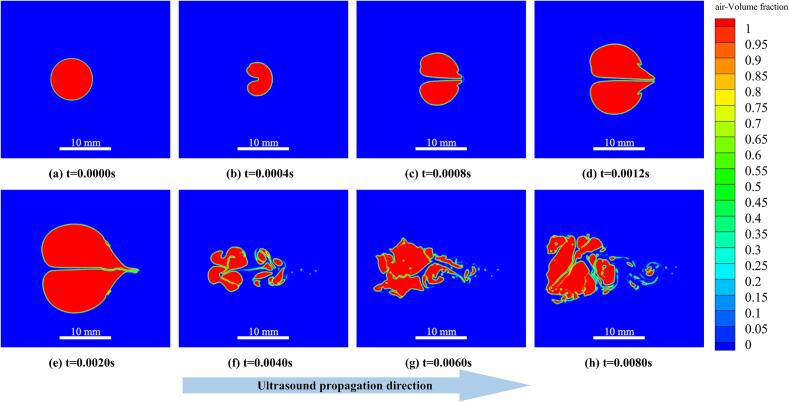
Temporal evolution of bubble collapse morphology induced by ultrasonic cavitation jets at 25 kHz and a vibration amplitude of 30 μm. Color contours denote the air volume fraction, with red regions representing the gas phase. The arrow indicates the ultrasound propagation direction.

To facilitate a clearer and more in-depth qualitative and quantitative analysis of the typical bubble collapse process, a representative case with an ultrasonic excitation frequency of f = 25 kHz (corresponding to an acoustic period of T = 40  μs) and a vibration amplitude of A = 30 μm was selected for detailed discussion. The air volume fraction contours shown in Fig. 3(a–h) illustrate the morphological evolution of the bubble within a simulated time window of 0.008 s (approximately 200 acoustic cycles at 25 kHz). The ultrasonic wave propagates from left to right, with the vibrating plate located on the left side of the fluid domain.

Based on the observed bubble morphology and flow-field characteristics, the collapse process can be divided into four typical stages: initial acoustic response, early asymmetric deformation and jet initiation, asymmetric contraction and jet intensification, and fragmentation with post-collapse dispersion.

**Fig. 4:**
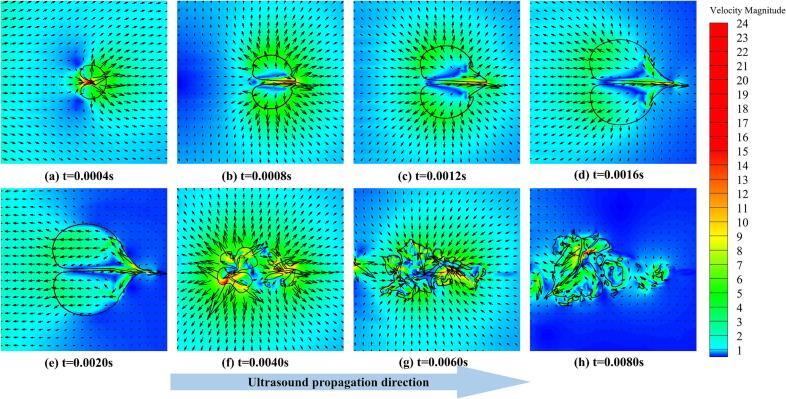
Velocity vector fields and velocity magnitude contours in the fluid domain at an ultrasonic frequency of 25 kHz and a vibration amplitude of 30 μm. The black solid line represents the bubble interface, and the arrows indicate the local flow velocity direction.

During the initial acoustic response stage (t = 0–0.0004 s), the bubble remains in a quasi-stable state and begins to respond to the applied ultrasonic field, as shown in Fig. 3(a) and (b). The bubble maintains an approximately spherical shape, and the interface remains smooth and intact, with no visible breakup or separation. As indicated in Fig. 4(a), the velocity field in the surrounding liquid is relatively uniform during this stage, and flow disturbances near the bubble are weak. The local velocity magnitude is mainly distributed in the range of 2–5 m/s. The periodic high-frequency mechanical vibration generated by the ultrasonic vibrating plate propagates through the liquid and induces cyclic pressure fluctuations in the flow field. These pressure variations tend to compress the bubble, and the acoustic pressure initially manifests as a small perturbation at the gas-liquid interface. As shown in Fig. 4(b), a slight increase in velocity can be observed near the bubble interface on the side facing the vibrating plate (left side of the bubble), indicating the onset of non-uniform pressure distribution at the gas-liquid interface due to ultrasonic wave propagation.

During the early asymmetric deformation and jet initiation stage (t = 0.0004–0.0020 s), the bubble interface develops increasingly evident non-uniform deformation under the action of ultrasonic cavitation pressure. As a result, the bubble gradually adopts a characteristic cashew-like shape, while its overall volume continues to increase. With the continued action of the ultrasonic field, the bubble reaches its maximum expansion at approximately t = 0.002 s, and a distinct penetrating jet structure begins to form locally inside the bubble, as shown in Fig. 3(c–e). Analysis of the velocity vector fields and velocity magnitude contours in Fig. 4(b–e) reveals that this stage still corresponds predominantly to the low-pressure-driven expansion response of the bubble. However, the interface evolution is no longer spatially symmetric. In particular, a pronounced velocity gradient develops on the side facing the vibrating plate, where local velocities reach approximately 15–20 m/s. This behavior indicates that, under the negative pressure half-cycle of the ultrasonic wave, the gas inside the bubble expands rapidly, while the surrounding liquid is accelerated away from the bubble interface and the early development of a directional microjet is already initiated.

During the asymmetric contraction and jet intensification stage (t = 0.0020–0.0040 s), the ultrasonic pressure transitions from the negative to the positive half-cycle, causing the bubble to rapidly shift from expansion to contraction. Owing to the presence of the vibrating wall and the spatially non-uniform distribution of acoustic pressure, the bubble interface on the side facing the vibrating plate collapses first. As a result, the bubble exhibits a pronounced asymmetric contraction. As shown in Fig. 4(e–f), the velocity magnitude near the interface on the vibrating-plate side increases sharply, with local velocities exceeding 20 m/s. The flow is directed normal to the interface and toward the bubble interior, forming a characteristic impact jet structure. Based on the velocity variation, the local fluid velocity near the interface increases by approximately 10 m/s within about 0.001 s, corresponding to an equivalent acceleration on the order of 10^4^ m/s^2^. Under the action of this strong impact jet, the bubble interface on the vibrating-plate side develops pronounced wrinkles and localized indentations (Fig. 3(f)). The interface stability deteriorates rapidly, and the jet continues to intensify as it penetrates through the bubble interior.

During the fragmentation and dispersion stage (t ≥ 0.0040 s), the bubble enters the late stage of collapse. Under the combined effects of strong impact loading and tearing by the asymmetric jet, the original bubble disintegrates. The bubble interface breaks into multiple small-scale structures, which rapidly disperse into the surrounding liquid. As shown in Fig. 3(f–h), the gas phase no longer maintains a continuous structure after breakup but instead forms multiple discrete gas clusters with highly irregular and dispersed morphologies. At t = 0.0040 s (Fig. 3(f)), the bubble outline has already split into at least three to four dominant gas clusters, whose characteristic sizes are significantly smaller than that of the initial bubble. At a later time of t = 0.0080 s (Fig. 3(h)), the gas phase distribution becomes even more dispersed, and interfacial instability intensifies markedly. This indicates that the bubble has undergone complete breakup followed by secondary refinement, occupying a much larger spatial region. Compared with the initial concentrated configuration, the overall dispersed gas-liquid interfacial area increases substantially. The corresponding velocity magnitude and vector fields shown in Fig. 4(g–h) further elucidate the flow characteristics during this stage. Multiple localized vortical structures and regions of intense flow disturbance emerge within the fragmentation zone, accompanied by highly disordered velocity directions. Although the overall peak velocity decreases after the primary collapse event, pronounced local velocity gradients persist. In some regions, the local velocity remains as high as approximately 13 m/s (orange regions in Fig. 4(g)). These complex flow features—including recirculation, rotational motion, and intersecting shear layers—indicate that ultrasonic cavitation-induced bubble fragmentation generates strong nonlinear turbulent effects, which promote the rapid dispersion and secondary refinement of the fragmented microbubbles.

The above analysis demonstrates that ultrasonic cavitation-induced bubble collapse is not limited to a purely structural breakup of the bubble. Instead, it is accompanied by intense evolution of the surrounding flow field, characterized by pronounced variations in velocity and vortical structures. The collapse process exhibits a clear stage-wise behavior, progressing sequentially from the initial acoustic response, and early asymmetric deformation and jet initiation, and asymmetric contraction and jet intensification, and fragmentation with post-collapse dispersion. Through this sequence, the cavitation-induced jet gradually completes the destruction of the bubble under ultrasonic excitation. Within the selected simulation window, the original parent bubble does not exhibit a simple rebound as an intact structure after the primary collapse. Instead, the later-stage behavior is mainly associated with the evolution of fragmented residual gas clusters.

The predicted asymmetric collapse toward the vibrating boundary and directional jet penetration share the general characteristics of near-wall bubble dynamics reported in previous experimental and numerical studies under ultrasonic excitation[23], [29], [35], [38]. In particular, the preferential collapse on the boundary-facing side and the formation of strong liquid jets are qualitatively consistent with the general trends reported for acoustically driven bubbles affected by wall constraints. These similarities provide additional confidence in the physical interpretation of the simulated collapse process and support the subsequent parametric analysis.

### 3.2 Effect of ultrasonic frequency on bubble collapse behavior

To investigate the effect of ultrasonic frequency on bubble collapse efficiency and cavitation behavior, numerical simulations were performed at a fixed vibration amplitude of A = 20  μm. Four representative ultrasonic frequencies (20, 25, 30, and 40 kHz) were selected. The bubble volume evolution, collapse morphology, and impact intensity under different frequencies were comparatively analyzed.

#### 3.2.1 Evolution characteristics of air volume fraction

**Fig. 5:**
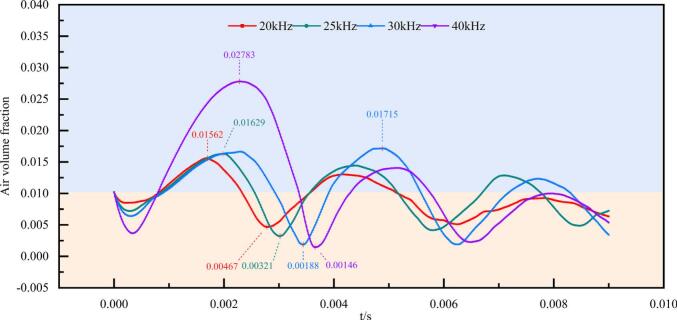
Temporal evolution of air volume fraction under different ultrasonic frequencies (20, 25, 30, and 40 kHz) at a fixed vibration amplitude of 20 μm.

Fig. 5 presents the temporal evolution of the air volume fraction within 0.009 s under different ultrasonic frequencies at a vibration amplitude of 20 μm. As shown, the air volume fraction exhibits pronounced nonlinear oscillations for all frequencies. These oscillations are characterized by rapid expansion followed by violent contraction, which is consistent with the typical dynamics of inertial cavitation bubbles. Specifically, the bubble expands during the negative pressure phase of the ultrasonic wave and collapses during the positive pressure phase.

During the initial expansion stage, significant differences in the peak air volume fraction are observed among the tested frequencies. At 40 kHz, the bubble exhibits the most intense expansion, with a peak air volume fraction of approximately 0.028, which is notably higher than those observed under the 20–30 kHz conditions (approximately 0.015–0.018). This result indicates that, under a fixed vibration amplitude, higher ultrasonic frequencies can substantially enhance the bubble expansion capability during the initial stage. This behavior can be attributed to the vibration velocity of the oscillating wall, which approximately follows $\mu_{0}\approx 2\pi fA$. A higher ultrasonic frequency corresponds to a larger instantaneous wall velocity and acoustic pressure amplitude, thereby strengthening the inertial driving force exerted by the liquid on the bubble interface.

Further analysis reveals that the occurrence time of the first pronounced expansion peak exhibits a clear delay with increasing ultrasonic frequency. Specifically, the first peak in air volume fraction appears at approximately 1.7, 1.9, 2.1–2.4, 2.3 ms for frequencies of 20, 25, 30, and 40 kHz respectively. This peak delay can be primarily attributed to the phase-lag behavior associated with forced bubble oscillations. For a bubble with an initial radius of approximately 4 mm, the natural resonance frequency is estimated to be around 3–5 kHz, which is far below the driving frequencies adopted in this study (20–40 kHz). As a result, the bubble oscillates in a strongly off-resonant forced regime. Under such conditions, the radial response of the bubble exhibits a significant phase lag relative to the external acoustic pressure.

To qualitatively interpret this effect, the phase relationship may be approximately expressed as

(11) $$\phi \left(\omega \right)=\arctan\left(\frac{2\delta \omega }{\omega_{0}^{2}-\omega^{2}}\right)$$

where ω is the driving angular frequency, $\omega_{0}$ is the natural angular frequency of the bubble and δ represents the overall damping coefficient[44]. When the driving frequency is much higher than the natural frequency of the bubble, the phase lag increases significantly, indicating that the bubble response is delayed relative to the external pressure excitation. Consequently, the bubble requires the accumulation of energy over multiple acoustic cycles before a pronounced expansion peak can occur. This trend is qualitatively consistent with classical bubble dynamics theory.

#### 3.2.2 Frequency dependence of bubble collapse morphology

**Fig. 6:**
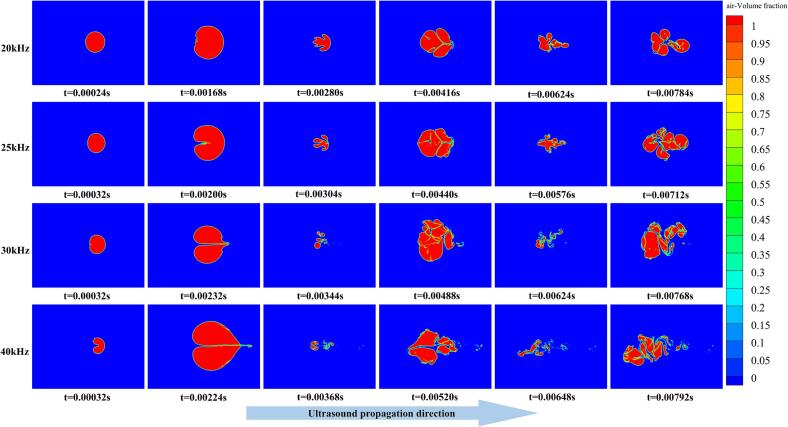
Temporal evolution of bubble collapse morphology under different ultrasonic frequencies at a vibration amplitude of 20 μm. Color contours denote the air volume fraction, with red regions representing the gas phase. The rows correspond to ultrasonic frequencies of 20–40 kHz.

Because the acoustic period varies with ultrasonic frequency, a direct comparison of bubble morphology at a single time instant cannot accurately capture the frequency effect. Therefore, based on the peak and trough moments of the air volume fraction curves for each frequency shown in Fig. 5, the corresponding bubble morphologies were extracted. The resulting collapse evolution characteristics under different frequencies are presented in Fig. 6.

As illustrated in Fig. 6, increasing ultrasonic frequency makes the bubble more prone to pronounced asymmetric deformation during both the expansion and subsequent compression stages, accompanied by the formation of stronger directional jets. In particular, under the 40 kHz condition, the bubble undergoes highly violent fragmentation during the first collapse event, producing a large number of small and dispersed microbubble clusters. This behavior indicates the strongest instantaneous collapse intensity among the tested frequencies.

At lower ultrasonic frequencies (20 and 25 kHz), the bubble retains a relatively intact main structure after the first collapse, experiencing only limited fragmentation and dispersion. During subsequent acoustic cycles, the primary bubble cluster continues to participate in repeated expansion and collapse processes, with fragmentation and re-coalescence occurring alternately. As a result, the overall dispersion degree remains relatively low. In contrast, at higher frequencies (30 and 40 kHz), the bubble almost completely disintegrates into numerous small fragments after the first compressive collapse. Under the 40 kHz condition in particular, the resulting microbubble cloud exhibits weak expansion in subsequent cycles. Some microbubbles rapidly decay due to diffusion and dissolution effects, making it difficult to re-establish effective residual bubble clusters. Consequently, the macroscopic cavitation activity is significantly weakened within a short time.

#### 3.2.3 Comparison of impact intensity and cavitation persistence

**Fig. 7:**
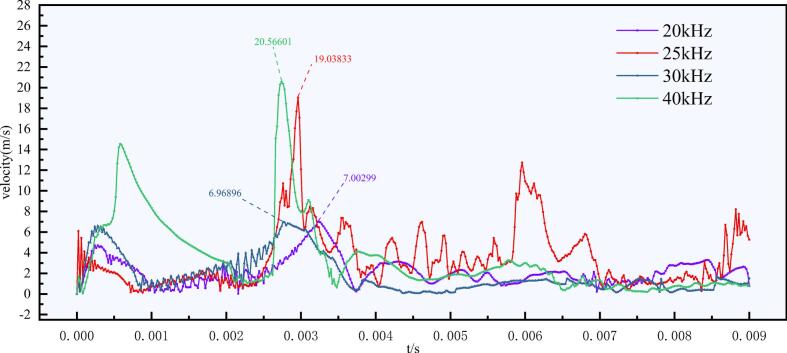
Temporal evolution of fluid velocity at the monitoring point under different ultrasonic frequencies at a vibration amplitude of 20 μm. Dashed lines and numerical labels indicate the peak velocity values.

To further quantify the impact effects during bubble collapse under different ultrasonic frequencies, the temporal variation of the fluid velocity at a monitoring point was analyzed at a fixed vibration amplitude, as shown in Fig. 7. The results indicate that the peak velocity does not increase monotonically with ultrasonic frequency. Under the 40 kHz condition, the maximum instantaneous velocity reaches 20.56 m/s at approximately 2.74 ms. For the 25 kHz case, a comparable peak velocity of 19.04 m/s occurs at around 2.96 ms. In contrast, the maximum velocities under 20 kHz and 30 kHz are only 7.01 m/s and 6.97 m/s, respectively. These results demonstrate that, within an appropriate frequency range, a moderate ultrasonic frequency can induce impact jets with intensities comparable to those generated at higher frequencies.

Significant differences are observed in both the instantaneous impact intensity and the persistence of the impact effect among different frequencies. Under the 40 kHz condition, the maximum instantaneous velocity reaches the highest value among all tested cases, indicating the strongest single-event jet impact. However, the velocity decays rapidly after the primary peak, and the subsequent fluctuations remain relatively weak, suggesting that the cavitation-induced impact is difficult to sustain for a long duration at this high frequency. In contrast, although the 25 kHz condition does not produce the highest instantaneous velocity peak, it still generates a comparably strong initial impact and exhibits more persistent post-peak velocity fluctuations over several subsequent acoustic cycles. This behavior indicates that the cavitation impact under 25 kHz is more sustained within the flow field.

Combined with the morphological analysis shown in Fig. 6, the results indicate that the effect of ultrasonic frequency is governed by a trade-off between instantaneous collapse intensity and cavitation persistence. Although 40 kHz produces the strongest single-event jet impact, it also promotes excessive fragmentation and rapid attenuation of residual bubble activity. By contrast, 25 kHz maintains a comparably strong initial impact while preserving more sustained post-collapse activity and moderately sized residual bubble clusters. Therefore, within the investigated parameter range, 25 kHz provides a more favorable balance between impact strength and cavitation persistence.

### 3.3 Mechanism of ultrasonic frequency effects on bubble fragmentation degree

To quantitatively characterize the degree of bubble fragmentation under different ultrasonic frequencies, the bubble perimeter was introduced as a metric to represent the interfacial generation induced by bubble breakup. This parameter was derived from the air volume fraction contours obtained during the bubble collapse process. Within the two-dimensional numerical framework, bubble deformation and fragmentation under cavitation lead to an increase in the gas-liquid interfacial area in three dimensions, which can be equivalently reflected by a pronounced increase in the bubble perimeter on a two-dimensional cross-section. Therefore, the bubble perimeter serves as an effective quantitative indicator for evaluating cavitation-induced fragmentation intensity and newly generated interfacial area.

**Fig. 8:**
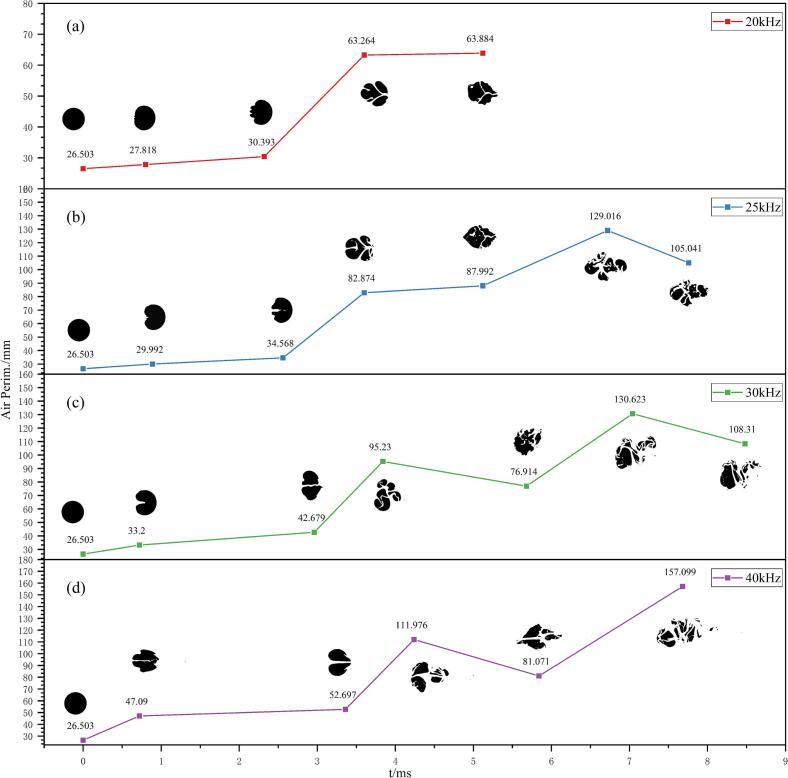
Temporal evolution of the cross-sectional perimeter of the bubble cloud during bubble collapse under different ultrasonic frequencies at a vibration amplitude of 20 μm. The cross-sectional perimeter represents the projected gas-liquid interfacial perimeter of the fragmented bubble cloud, and the black silhouettes show the corresponding instantaneous morphologies. Panels (a)–(d) correspond to 20–40 kHz.

Based on the temporal evolution of air volume fraction under different ultrasonic frequencies shown in Fig. 5, key time instants were selected at which the air volume was comparable to the initial bubble volume for each frequency. The corresponding air volume fraction contours were then extracted and analyzed. First, the contour images were binarized, where the black regions represent the deformed and fragmented bubble domains. Subsequently, automatic boundary detection was applied to the binarized images to calculate the total perimeter of the bubble cloud. The temporal variation of the bubble perimeter and the corresponding binarized fragmentation morphologies under different frequencies are presented in Fig. 8.

From the overall trend, the degree of bubble fragmentation under ultrasonic cavitation increases markedly with increasing ultrasonic frequency. Under low-frequency conditions, the fragmentation process is relatively mild, and the bubble perimeter exhibits only a limited increase over time, indicating that bubble deformation and a small number of breakup events dominate the process. For example, at 20 kHz (Fig. 8(a)), the bubble splits into only a few large fragments after the initial deformation stage, and the perimeter stabilizes after reaching its first peak. This behavior suggests that, under low-frequency excitation, the cavitation-induced impact intensity is insufficient to cause extensive fragmentation, and the bubble retains a relatively intact overall structure.

When the ultrasonic frequency increases to 25 and 30 kHz (Fig. 8b–c), bubble fragmentation becomes markedly more pronounced. In these cases, the bubble perimeter exhibits a more significant increase with time and reaches higher peak values in subsequent acoustic cycles, indicating the generation of a larger amount of newly formed gas-liquid interface. The air volume fraction contours show that the bubble breaks into multiple fragments of intermediate size, resulting in a more dispersed structure with substantially increased interfacial complexity. A comparison between the two frequencies reveals that, at 25 kHz, the bubble fragments are more uniformly distributed and the bubble cloud exhibits better dispersion. At 30 kHz, although the fragmentation intensity is further enhanced, relatively large fragment clusters can still be observed locally, and the uniformity of fragment size distribution is slightly reduced. This suggests that, in the medium-to-high frequency range, a further increase in frequency does not necessarily lead to a more favorable fragmentation structure.

At the higher frequency of 40 kHz (Fig. 8(d)), bubble fragmentation exhibits a highly transient character. The bubble perimeter increases rapidly within a short time and reaches the maximum value among all tested frequencies, indicating that a single cavitation event can generate the largest gas-liquid interfacial area. This observation is consistent with the significantly increased jet peak velocities obtained under high-frequency excitation. However, the air volume fraction contours show that, at 40 kHz, the bubble is excessively fragmented into a large number of very small microbubbles. The resulting bubble cloud decays rapidly during subsequent ultrasonic cycles and fails to form stable bubble nuclei, thereby shortening the effective duration of cavitation activity. Such excessive fragmentation limits the sustained utilization of the gas-liquid interface over time.

By integrating the evolution of bubble perimeter, air volume fraction morphology, and velocity peak characteristics, it is evident that the effect of ultrasonic frequency on bubble fragmentation is governed by a trade-off between instantaneous fragmentation intensity and cavitation persistence. At low frequency (20 kHz), fragmentation is insufficient and interfacial generation is limited. Although the fragmentation intensity at 30 kHz exceeds that at 25 kHz, the poorer uniformity of fragment size distribution does not further improve the overall fragmentation structure. At high frequency (40 kHz), the strongest instantaneous fragmentation is achieved, but excessive breakup leads to rapid attenuation of cavitation activity.

In contrast, at a fixed vibration amplitude of 20 μm, the 25 kHz condition exhibits a clear overall advantage. It enables effective initial bubble breakup driven by strong liquid jets, sustains a sufficient gas-liquid interface over multiple acoustic cycles, and produces bubble fragments with moderate and relatively uniform sizes. Consequently, a favorable balance is achieved among cavitation intensity, interfacial generation, cavitation persistence, and fragmentation uniformity, making 25 kHz a rational frequency choice for ultrasonic cavitation-enhanced bubble fragmentation.

### 3.4 Effect of vibration amplitude on bubble collapse behavior

Based on the preceding analysis, an ultrasonic frequency of 25 kHz exhibits a favorable balance between bubble collapse intensity and cavitation persistence. To further investigate the influence of vibration amplitude on cavitation-induced bubble collapse, four representative amplitudes (5, 10, 20, and 30 μm) were examined under the same ultrasonic frequency. Numerical simulations were performed to analyze the bubble morphological evolution, volume response, and impact intensity under different amplitude conditions.

**Fig. 9:**
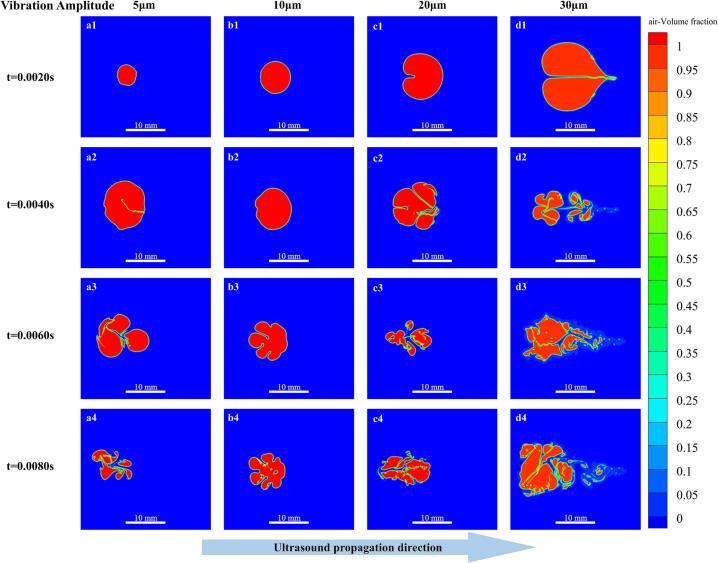
Temporal evolution of bubble collapse morphology under different vibration amplitudes at an ultrasonic frequency of 25 kHz. Color contours denote the air volume fraction, with red regions representing the gas phase. Columns correspond to vibration amplitudes of 5–30 μm.

As shown in Fig. 9, increasing the vibration amplitude causes the bubble collapse behavior to transition from mild deformation to intense nonlinear breakup under ultrasonic cavitation. This trend is consistent with the bubble dynamics described by the Rayleigh–Plesset equation[45], in which bubble radius evolution is governed by acoustic pressure amplitude, surface tension, and viscous damping. In particular, the acoustic pressure amplitude $P_{a}$ is positively correlated with the vibration amplitude A, and increasing amplitude significantly enhances the nonlinear oscillatory response of the bubble.

At a low vibration amplitude of 5 μm (Fig. 9(a1–a4)), the bubble mainly undergoes slow deformation and limited compression, while maintaining a relatively intact structure without obvious fragmentation. This behavior indicates that the cavitation intensity is insufficient to trigger violent collapse. When the amplitude is increased to 10 μm (Fig. 9(b1–b4)), the collapse rate increases noticeably, and asymmetric compression accompanied by initial fragmentation begins to occur. The bubble gradually splits into a dominant primary bubble and a small number of microbubbles, reflecting the enhancement of transient cavitation effects. Further increasing the amplitude to 20 μm (Fig. 9(c1–c4)) leads to pronounced deformation and necking at an earlier stage, followed by complete breakup into multiple discrete fragments. Under this condition, the collapse behavior gradually shifts from oscillation-dominated to collapse-dominated dynamics. At the highest amplitude of 30 μm (Fig. 9(d1–d4)), the bubble undergoes rapid and nearly instantaneous disintegration, producing numerous fine fragments and elongated tails. This highly nonlinear collapse behavior indicates that the acoustic pressure peaks exceed the cavitation threshold, thereby triggering cascade collapse and secondary cavitation processes.

From a dynamical perspective, the bubble collapse rate exhibits a pronounced positive correlation with vibration amplitude. This trend is consistent with the scaling law of ultrasonic cavitation theory, in which the acoustic intensity follows $I\propto A^{2}f^{2}$. In the low-amplitude range (5–10 μm), the bubble response remains predominantly oscillation-dominated, with relatively weak collapse intensity and only limited fragmentation. When the amplitude increases to 20–30 μm, the cavitation mode shifts to transient cavitation, accompanied by a marked enhancement in fragmentation intensity. Correspondingly, the bubble collapse time is reduced from above approximately 0.008 s to below 0.006 s. With increasing amplitude, the local acoustic pressure gradient and the Bjerknes force are significantly intensified, leading to enhanced interfacial instability and accelerated asymmetric bubble collapse. The asymmetric deformation of the bubble toward the vibrating plate observed in the simulations is consistent with the direction of acoustic wave propagation. Moreover, since the operating frequency exceeds the Minnaert resonance frequency of the bubble, the bubble response exhibits a typical subharmonic resonance behavior.

**Fig. 10:**
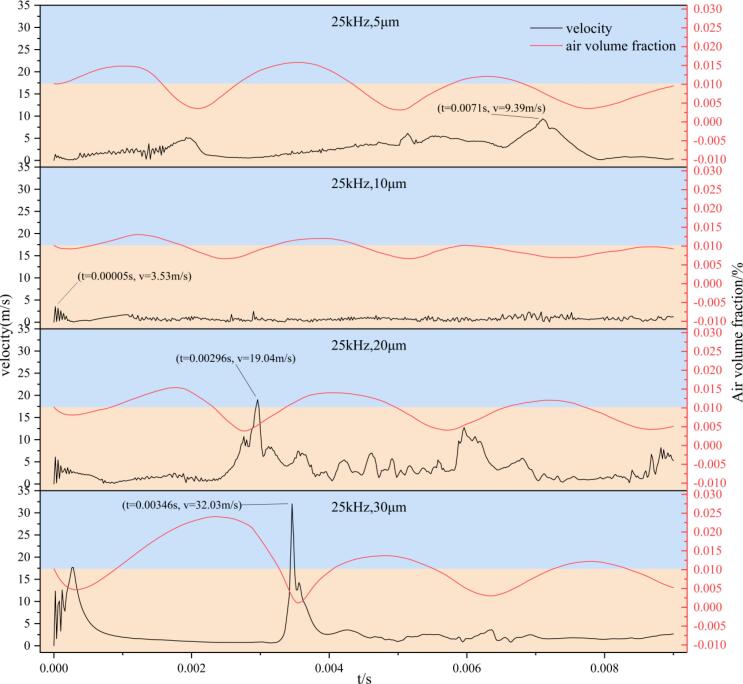
Temporal evolution of fluid velocity and air volume fraction at the monitoring point under different vibration amplitudes at an ultrasonic frequency of 25 kHz. The black curves represent the local fluid velocity (left axis), while the red curves denote the air volume fraction (right axis). From top to bottom, the panels correspond to vibration amplitudes of 5, 10, 20, and 30 μm, respectively. The numerical labels indicate the peak velocity values and their corresponding time instants.

To further quantify the influence of vibration amplitude on bubble dynamics, Fig. 10 presents the temporal evolution of the air volume fraction and the fluid velocity at the monitoring point under different amplitude conditions.

The peak air volume fraction reflects the maximum bubble expansion volume and shows an overall increasing trend with amplitude. This indicates that higher amplitudes provide stronger negative pressure driving, enabling the bubble to expand to larger sizes and store more energy. The velocity histories at the monitoring point further reveal differences in collapse impact intensity among the tested amplitudes. In all cases, the velocity peaks occur when the bubble is compressed to its minimum volume, corresponding to the formation of liquid jets and the onset of impact. At an amplitude of 5 μm, the maximum local velocity reaches approximately 9.39 m/s, indicating the formation of a jet with moderate impact strength. Under the 10 μm condition, the peak velocity decreases to about 3.52 m/s, suggesting that the collapse energy is not effectively focused. When the amplitude increases to 20 μm and 30 μm, the peak velocities rise sharply to approximately 19.04 m/s and 32.03 m/s, respectively, corresponding to the stages of initial and intense bubble collapse. These results demonstrate that, under high-amplitude excitation, the released energy can locally induce strong shock waves and high-speed microjets, which serve as the dominant mechanisms driving rapid bubble fragmentation and dispersive collapse.

The persistence of cavitation-induced impact effects shows pronounced differences among high-amplitude conditions. At an amplitude of 20 μm, although the initial impact intensity is lower than that at 30 μm, multiple pronounced velocity fluctuations are still observed at the monitoring point after the primary peak. This indicates that cavitation events can be sustained within the fluid domain. After the initial collapse, residual bubbles continue to undergo further fragmentation and participate in subsequent acoustic cycles, thereby maintaining cavitation activity.

In contrast, at an amplitude of 30 μm, the fluid velocity reaches the highest instantaneous peak among all tested cases, indicating the strongest single-event collapse intensity. However, the velocity decays rapidly afterward and remains at a relatively low level during the later stage. This behavior suggests that the bubble is excessively fragmented during the first intense collapse, leading to a rapid attenuation of effective residual bubble activity.

Therefore, increasing vibration amplitude can significantly enhance the instantaneous impact intensity of bubble collapse, but excessively high amplitudes are less favorable for sustaining cavitation activity over time. Within the investigated parameter range, 20 μm does not correspond to the strongest instantaneous impact, but it provides a more favorable balance between strong initial collapse and sustained post-collapse cavitation response. Therefore, within the present parameter range, 20 μm can be regarded as a more balanced operating condition for ultrasonic-induced bubble breakup.

## 4 Conclusions

In this study, the sonophysical mechanisms governing bubble fragmentation near an active vibrating boundary were investigated through a high-resolution VOF numerical framework. By systematically investigating the coupling between acoustic parameters and interfacial dynamics, this work reveals the fundamental trade-offs in boundary-modulated ultrasonic cavitation. The primary findings are summarized as follows:

(1) Under ultrasonic excitation, bubble collapse is dominated by pronounced asymmetric contraction toward the vibrating wall and the formation of high-speed penetrating microjets. These microjets act as the primary driving mechanism for bubble breakup and fragmentation, leading to a substantial increase in gas-liquid interfacial complexity. The collapse process exhibits a clear stage-wise evolution, including initial acoustic response, early asymmetric deformation and jet initiation, asymmetric contraction and jet intensification, and fragmentation with post-collapse dispersion.
(2) Within the investigated frequency range, increasing ultrasonic frequency intensifies asymmetric collapse, jet strength, and fragmentation degree. However, the overall effectiveness is governed by a trade-off between instantaneous collapse intensity and cavitation persistence. Compared with insufficient fragmentation at low frequencies and excessive fragmentation at high frequencies, an ultrasonic frequency of 25 kHz provides a more favorable balance by inducing effective asymmetric collapse and jet impact while preserving moderately sized bubble clusters with relatively uniform fragment distributions. This balance enables simultaneous enhancement of fragmentation intensity, interfacial generation, and cavitation sustainability.
(3) Ultrasonic vibration amplitude plays a critical role in regulating cavitation energy input and impact intensity. As the amplitude increases, bubble collapse accelerates and fragmentation becomes more pronounced. In the low-amplitude range, the bubble response remains predominantly oscillation-dominated, whereas higher amplitudes promote stronger collapse and more evident fragmentation. Nevertheless, excessively high amplitudes lead to rapid energy dissipation and reduced cavitation persistence. Within the investigated range, an amplitude of 20 μm provides a more favorable balance between strong collapse intensity and sustained cavitation activity.

Overall, the combination of 25 kHz frequency and 20 μm amplitude represents a favorable operating regime within the investigated range. Under this condition, a better balance is achieved between jet-induced breakup intensity and the persistence of post-collapse bubble activity. These findings provide mechanistic insight into ultrasonic-induced bubble fragmentation near vibrating boundaries and offer useful guidance for ultrasonic-assisted wet dust-removal processes.

## CRediT authorship contribution statement

**Zhitao Zhao:** Writing – review & editing, Writing – original draft, Software, Formal analysis, Data curation. **Wei Lu:** Writing – review & editing, Funding acquisition, Conceptualization. **Qingsong Zhang:** Writing – review & editing, Supervision. **Hui Zhuo:** Writing – review & editing, Supervision. **Zhaoyang Su:** Writing – review & editing, Funding acquisition.

## Declaration of competing interest

The authors declare that they have no known competing financial interests or personal relationships that could have appeared to influence the work reported in this paper.
